# Coincidental Central Precocious Puberty and Wilms Tumor in a 5-Year-Old Girl

**DOI:** 10.1155/2019/5427207

**Published:** 2019-09-08

**Authors:** Laura Kasongo, Patricia Forget, Ramona Corina Nicolescu

**Affiliations:** ^1^Department of Pediatrics, University of Liege, Centre Hospitalier Régional Citadelle Liege, Blvd du 12eme de Ligne nr 1, 4000 Liège, Belgium; ^2^Department of Pediatric Hematology and Oncology, University of Liege, Centre Hospitalier Régional Citadelle Liege, Blvd du 12eme de Ligne nr 1, 4000 Liège, Belgium; ^3^Pediatric Endocrinology Outpatient Clinic, Valdor Isosl Hospital, Rue Basse-Wez 145, 4020 Liège, Belgium

## Abstract

Wilms tumor is the most frequent pediatric renal malignancy, and its usual presentation is an abdominal mass or hematuria. Unusual presentations have also been reported, such as paraneoplastic syndromes (acquired von Willebrand disease, sudden death due to pulmonary embolism, and Cushing syndrome). These conditions can precede, occur concomitantly, or present in a later phase of tumor development. Precocious puberty, as paraneoplastic endocrine syndrome, has already been described in children with malignant tumors (brain, gonadal, adrenal tumors, and hepatoblastoma). However, little is known about central precocious puberty, as paraneoplastic manifestation of nephroblastoma or secondary to its specific chemotherapy. Here, we report a case of Wilms tumor and simultaneous precocious puberty in a 5-year-old girl. The initial diagnosis was premature telarche, but the clinical and biological pubertal progression changed our diagnosis to idiopathic central precocious puberty. Chemotherapy and nephrectomy were well tolerated, and we began treatment with a gonadotropin-releasing hormone agonist which showed favorable outcomes over the short term. We highlight the need for early diagnosis and work-up in all patients of precocious puberty, in order to institute timely management.

## 1. Introduction

Premature telarche (PT), a benign condition in girls younger than 8 years of age, defines an isolated breast development, without other signs of puberty. The diagnosis is usually made clinically and does not require biochemical or radiological tests. The most consistent aspect of PT management requires serial monitoring of the patient to ensure the continued absence of other sexual characteristics. The spontaneous regression of breast enlargement is usually the main evolution rule. After this self-limited event, puberty usually occurs and progresses physiologically.

However, it is possible that PT progresses to precocious puberty (PP), with pubertal initiation before the age of 8 years in girls and 9 years in boys. The incidence of PP was recently estimated to be 0.2% in girls and 0.05% in boys [1]. Approximately 90% of girls and 25–60% of boys, with PP have an idiopathic cause [2, 3]. The organic etiology is more frequently in boys and includes congenital and acquired cranial and extracranial lesions.

PP is one of the most common endocrine-related manifestations of neoplasia in pediatric patients. Other systemic or endocrine manifestations, globally referred to as paraneoplastic syndrome, include hypercalcemia, inappropriate antidiuretic hormone secretion, ectopic adrenocorticotrophic hormone secretion, and hypoglycemia. Endocrine abnormalities can also appear subsequently, after the neoplasia has been treated, associated with drugs or radiation.

Herein, we present the case of a child with Wilms tumor and concomitant PT who progressed to central PP (CPP). In addition, we provide a review of the literature relating to neoplasia-associated puberty and paraneoplastic manifestations of renal tumors.

## 2. Case Presentation

A previously healthy 5-year-old girl of African origin presented to the Emergency Department (ED) with macroscopic hematuria appeared 24 hours before presentation and isolated abdominal pain for one month previously. There was no history of fever, vomiting, urinary or systemic symptoms, weight loss, or abdominal trauma.

The patient had no significant medical history, in particular, head injuries such as trauma, hemorrhage, or infection, and did not take any medications. Family history was unremarkable, with no antecedents of neoplasm or precocious puberty. Her mother was not taking any contraceptive hormonal medications, and there was no history of any other source of exposure to hormonal drugs.

On physical examination, the child appeared well. Her anthropometric parameters were as follows: weight 21 kg (50^th^ percentile); height 117 cm (50–75^th^ percentile). Her vital signs were normal, except for blood pressure at 130/90 mm Hg (>99^th^ percentile for sex and height). She had neither dysmorphic features nor skin lesions (café-au-lait spots, facial acne). On abdominal examination, we palpated a mass, measuring 6 cm with regularly margins in the right upper quadrant. There was no central nervous system dysfunction (headache, vomiting or visual abnormalities). She had bilateral, symmetrical nontender enlargement of breast buds corresponding to stage 2 of the Tanner classification. There was no pubic or axillary hair, and her thyroid was normal on clinical inspection.

Laboratory work-up in the ED included a complete blood count, metabolic panel, and urinalysis. Her hematologic and basic metabolic panel, serum electrolytes, and liver and renal function tests were normal. Urinalysis confirmed hematuria. The child underwent an ultrasonography, which revealed a well-circumscribed heterogeneous echogenic mass, measuring 9.2 × 9.5 cm, in the right renal fossa. With suspicion of Wilms tumor, the child was admitted into the Oncology Department. On abdominal and pulmonary computerized tomography (CT), a high suspicion of nephroblastoma was made. Histological examination confirmed the diagnosis of intermediary-risk nephroblastoma stage I.

The first endocrine work-up revealed a normal thyroid function and prepubertal gonadotropin levels (see Table 1). Ultrasonography excluded ovarian and adrenal masses and showed infantile ovaries and uterus. Bone age was not performed. The endocrine diagnosis was bilateral PT.

The International Society of Pediatric Oncology (SIOP) 2001 chemotherapy protocol (dactinomycin/vincristine) was commenced, with complete excision of the tumor performed 4 weeks later. Additional chemotherapy cycles were provided after surgery, for a total duration of 3 months. The clinical tolerance and response were excellent, without any side effects or signs of residual tumor.

A second endocrine assessment was performed at the end of chemotherapy. Surprisingly, we identified an accelerated linear growth (a gain of 3 cm in 4 months) in the context of rapid breast development (passage from stage 2 to stage 3 over 4 months) with no axillary or pubic hair. The second hormonal work-up found an activated pituitary-gonadal axis (Table 1) along with advanced skeletal maturation and ultrasound signs of uterine hormonal impregnation. A gonadotropin-releasing hormone analog (GnRHa) stimulation test was carried out and showed a pubertal response, confirming gonadotropin-dependent puberty (Table 1).

**Figure 1:**
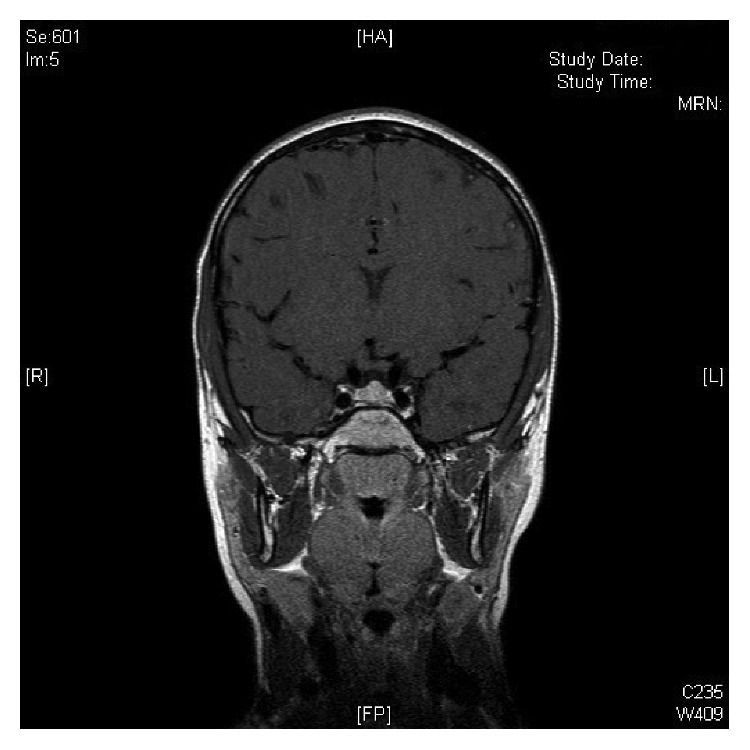
Coronal post-Gadolinium brain MRI image showing a normal pituitary morphology, of pubertal size, with marked upper surface convexity.

Magnetic resonance imaging (MRI) of the brain showed a morphologically normal pituitary, but of pubertal size (pituitary height of 6 mm and marked convexity of the upper surface) and no congenital or acquired lesions in the pineal or hypothalamic-optic region (Figure 1). Human chorionic gonadotropin (hCG) levels (repeated twice at different time points) were normal. hCG-secreting tumor was considered as potential etiology of PP, but this diagnosis was excluded on both biological (high LH and FSH levels, normal hCG) and radiological arguments (no tumor in liver, mediastinum, ovaries, or CNS).

The definitive diagnosis of idiopathic CPP was made, and we began treatment with GnRHa (11.25 mg every 3 months, intramuscularly). Six months later, we noted a reassuring clinical evolution, with partial regression of breast development, reduction in growth velocity (2 cm/6 months), and increasingly random LH level at 3.4 IU/L. Recently, a prospective study [4] showed that this biological evolution, with incomplete suppression of LH levels, is common in children with CPP under treatment. In a patient with a favorable clinical response (neither clinical pubertal progression, nor an advancement of bone age), this biological evolution should not be interpreted as indicative of treatment failure.

The global evolution of our patient, from oncological diagnosis up to 6 months after the beginning of CPP treatment, is depicted in Table 1.

## 3. Discussion

At the time of oncological diagnosis, our patient presented with classical PT. Her subsequent evolution, with accelerated height velocity (3 cm/4 months) in the context of somatic stress related to nephroblastoma treatment (nephrectomy and chemotherapy), was the first finding signaling the evolution to PP.

PT cases progressing to PP are described in the literature, with a prevalence of 9 to 14% [5, 6]. Neither clinical nor biological findings were suggestive of a progression although a recent retrospective study analyzing the etiology, MRI abnormalities, and growth velocity of girls with PP found that growth velocity can effectively differentiate PP from PT in girls aged 6–8 years [7]. In another study [8], similar elements were found, with a higher height and bone age/chronological age ratio as risk factors for progression to PP.

The diagnosis of PP requires a clinical, biological, and radiological evaluation. A detailed personal and family history and a complete physical examination including the assessment of linear growth and secondary sexual features (breast development in girls and testis measurement in boys, and pubic hair development in both sexes) and their classification on the basis of the Marshall and Tanner are necessary information. Bone age (typically advanced) and pelvic ultrasound (uterine and ovarian dimensions) are well correlated. The diagnostic value of basal LH is variable, and in this circumstance, the gold standard of the biochemical diagnosis is based on the assessment of gonadotropins, mainly LH, after stimulation with GnRHa. A peak stimulated LH of >5 IU/L along with an LH/FSH ratio of ≥2 are consistent with the diagnosis of CPP [9]. However, some challenges in the hormonal assessment of children with sexual precocity at the time of diagnosis still remain.

Wilms tumor is known to be associated with paraneoplastic systemic findings (hypertension, erythrocytosis, and acquired von Willebrand disease) and endocrine manifestations including hypercalcemia, Cushing syndrome, and hypoglycemia [10–12].

Given the troublesome presentation of our case, we reviewed the literature on neoplasm-related PP and effects of cancer therapy upon pubertal activation. Brain, gonadal, adrenal tumors, and hepatoblastoma are the most frequent tumors that may be associated with PP [13].

CNS tumors can disrupt hypothalamic and pituitary function, with secondary CPP. The most common tumors are hypothalamic (hamartomas, gliomas, and germinomas), chiasmatic (gliomas), and pineal (parenchymal tumors and germinomas) [14]. Ependymomas (located in the lateral posterior fossa) often cause obstructive hydrocephalus that leads to detrimental effects within the hypothalamic region, including precocious pubertal onset. A variety of mechanisms have been reported to activate the hypothalamus/pituitary axis, but these have yet to be fully elucidated. One of the first pathophysiological hypotheses was related to tumor location, which is seen as a major contributing factor in the premature activation of the hormonal axis [14]. More recently, a functional hypothesis, stating that tumor cells can produce hormones or substances which subsequently interfere with physiological GnRH secretion and GnRH neuron differentiation, has become more accepted. In boys, germinal cell tumors that produce hCG, a glycoprotein in which the *α*-subunit is identical to that of LH, induce precocious puberty due to cross reaction between hCG and the LH receptor. The clinical profile of this condition results in pubertal changes to the external genitalia, but with less testicular enlargement (secondary to the lack of Sertoli cell stimulation from FSH). The biological profile includes low levels of LH/FSH and high levels of testosterone due to the stimulatory effect of hCG on the LH receptor in the testes. Final diagnosis is made by measuring serum and cerebrospinal fluid hCG levels. Pediatric germ-cell tumors that induce this type of gonadotropin-dependent precocious puberty can also be mediastinal (particularly in Klinefelter syndrome) [15, 16], hepatic, or gonadal.

In females, hCG-secreting tumors can induce PP by several possible mechanisms, including the cosecretion of hCG and estradiol, low FSH activity of the very high hCG, and high tumoral aromatase activity for some cranial dysgerminomas [17–19].

The risk of organic pathology, particularly intracranial, is much higher in boys than in girls [20]. Age seems to be an important predictor of cerebral abnormalities (the younger the child, the higher the risk), and such lesions can be evaluated efficiently with MRI imaging.

Cranial radiation therapy (CRT) for cerebral tumors, with high radiation doses, has direct effects on GnRH with clinical and biological CPP. CPP is among the most common complications that can occur following CNS tumors and/or CRT affecting the hypothalamus/pituitary [21]. The risk of developing early puberty is greater for younger children [22], and the proposed mechanism of hypothalamic/pituitary dysfunction caused by irradiation is the disinhibition of cortical influences upon the hypothalamus.

Other types of tumors that are able to induce PP include hepatoblastomas and gonadal neoplasia. A hepatoblastoma is a hepatic type of tumor which can produce hCG. This form of tumor is not uncommonly associated with PP in males (testicular stimulation by hCG). Some tumor cells are also able to secrete testosterone [22]. Gonadal tumors are associated with PP due to excessive sex hormone secretion by the organ containing the tumor, or the tumor's tissue of origin. For example, adrenocortical tumors are associated with PP because of excessive androgen production [13].

An interesting point to consider is whether the CPP in our case represented a coincidental finding. We performed a full review of the published literature indexed in PubMed up to January 2019 regarding Wilms tumor and puberty. Search terms included “Wilms tumor,” “paraneoplastic syndrome,” and “precocious puberty.” Our search failed to identify existing reports relating to the coexistence of Wilms tumor and CPP.

It is possible that the pathogenesis of CPP could be related to the concomitant effect of chemotherapy along with functional alterations in the HPG axis, but none of the existing literature described similar circumstances. Chemotherapy could cause some anterior pituitary dysfunction in the survivors of childhood cancer, with hormonal deficiencies but not activation of the hypothalamic/pituitary axis. Alkylating agents, such as cyclophosphamide, lomustine, and procarbazine, are known to cause premature ovarian insufficiency and can indirectly affect pituitary function, but this adaptive pituitary response is not considered a disease [23].

Collating data from the literature review relating to the proposed pathophysiological mechanisms underlying paraneoplastic syndrome and neoplasia-induced pubertal activation and considering this alongside our patient's clinical evolution, we finally established that the association between Wilms tumor and CPP was purely coincidental.

## 4. Conclusion

We report a case of PT with rapid progression to CPP in a 5-year-old girl with a concomitant Wilms tumor. Our observations may shed light on the necessity to diagnose PP early in every child irrespective of the presenting illness. A complete clinical examination and prompt referral to a pediatric endocrinologist for specific work-up and treatment are helpful in limiting early and late long-term health implications (short stature, psychosocial outcomes, metabolic and cardiac events, and the risk of breast cancer development).

We therefore recommend that when faced with PP in a child with a concomitant tumor, the diagnosis of paraneoplastic or neoplasia-associated PP should be discussed.

## Figures and Tables

**Table 1:** Patient's evolution.

	At admission	4 months later	10 months later
Clinical data	Weight 21 kg Height 117 cm Tanner stage II-breast development	Weight 21 kg Height 120 cm (+3 cm/4 months) Tanner stage III-breast development	Tanner stage II-breast development

Hormonal data	LH < 0.3 IU/L (normal range <0.5) FSH 0.9 IU/L (normal range <1) Estradiol < 20 ng/L (normal range <25)	LH 1.4 IU/L FSH 3.8 IU/L Estradiol 54 ng/L hCG < 2 U/L (normal range <2) LH (40 min post 100 *µ*g triptorelin injection, sc) 13 IU/L FSH (40 min after 100 *µ*g triptorelin injection, sc) 6.3 IU/L	LH 3.4 IU/L

Radiological data	Abdominal US well-circumscribed heterogeneous echogenic mass of 9.2 × 9.5 cm in right renal fossa	Bone age—8 years Pelvic US—normal ovary size and morphology, enlarged uterus with maximum length of 45 mm	
		Brain MRI—Pituitary height of 6 mm and marked convexity of the upper surface. No lesions in the pineal or hypothalamic-optic region	

Oncological diagnosis and treatment	Wilms tumor (nephroblastoma)	End chemotherapy	
	SIOP WT01 protocol		
	Right nephrectomy		

Endocrine diagnosis and treatment	PT No treatment	CPP GnRHa 11.25 mg intramuscularly every 3 months	Same treatment

LH: luteinizing hormone; FSH: follicular stimulating hormone; sc: subcutaneous; US: ultrasound; hCG: human chorionic gonadotropin; SIOP: International Society of Pediatric Oncology; PT: premature telarche; CPP: central precocious puberty; GnRHa: gonadotrophin releasing hormone analog.

## References

[1] Teilmann G., Pedersen C. B., Jensen T. K., Skakkebaek N. E., Juul A. (2005). Prevalence and incidence of precocious pubertal development in Denmark: an epidemiologic study based on national registries. *Pediatrics*.

[2] Latronico A. C., Brito V. N., Carel J.-C. (2016). Causes, diagnosis, and treatment of central precocious puberty. *The Lancet Diabetes & Endocrinology*.

[3] Kaplowitz P., Bloch C., The Section on Endocrinology (2016). Evaluation and referral of children with signs of early puberty. *Pediatrics*.

[4] Lewis K. A., Eugster E. A. (2013). Random luteinizing hormone often remains pubertal in children treated with the histrelin implant for central precocious puberty. *The Journal of Pediatrics*.

[5] Pasquino A. M., Pucarelli I., Passeri F., Segni M., Mancini M. A., Municchi G. (1995). Progression of premature thelarche to central precocious puberty. *The Journal of Pediatrics*.

[6] Bizzarri C., Spadoni G. L., Bottaro G. (2014). The response to gonadotropin releasing hormone (GnRH) stimulation test does not predict the progression to true precocious puberty in girls with onset of premature thelarche in the first three years of life. *The Journal of Clinical Endocrinology & Metabolism*.

[7] Varimo T., Huttunen H., Miettinen P. J. (2017). Precocious puberty or premature thelarche: analysis of a large patient series in a single tertiary center with special emphasis on 6- to 8-year-old girls. *Frontiers in Endocrinology*.

[8] Çiçek D., Savas-Erdeve S., Cetinkaya S., Aycan Z. (2018). Clinical follow-up data and the rate of development of precocious and rapidly progressive puberty in patients with premature thelarche. *Journal of Pediatric Endocrinology and Metabolism*.

[9] Chen M., Eugster E. A. (2015). Central precocious puberty: update on diagnosis and treatment. *Pediatric Drugs*.

[10] Wang J., Zhang G. (2008). Paraneoplastic cushing syndrome because of corticotrophin-releasing hormone-secreting Wilms’ tumor. *Journal of Pediatric Surgery*.

[11] Lee M. H., Choa U., Lee J.-W. (2014). Cushing syndrome secondary to CRH-producing Wilms tumor in a 6 year old. *Journal of Pediatric Endocrinology and Metabolism*.

[12] Segers H., van der Heyden J. C., van den Akker E. L. T., de Krijger R. R., Zwaan C. M., van den Heuvel-Eibrink M. M. (2009). Cushing syndrome as a presenting symptom of renal tumors in children. *Pediatric Blood & Cancer*.

[13] Wendt S., Shelso J., Wright K., Furman W. (2014). Neoplastic causes of abnormal puberty. *Pediatric Blood & Cancer*.

[14] Stephen M., Zage P., Waguespack S. (2011). Gonadotropin-dependent precocious puberty: neoplastic causes and endocrine considerations. *International Journal of Pediatric Endocrinology*.

[15] Völkl T. M. K., Langer T., Aigner T. (2006). Klinefelter syndrome and mediastinal germ cell tumors. *American Journal of Medical Genetics Part A*.

[16] Bowden S. A., Germak J. A. (2006). Klinefelter syndrome presenting with precocious puberty due to a human chorionic gonadotropin (hCG)-producing mediastinal germinoma. *Journal of Pediatric Endocrinology and Metabolism*.

[17] Kubo O., Yamasaki N., Kamijo Y., Amano K., Kitamura K., Demura R. (1977). Human chorionic gonadotropin produced by ectopic pinealoma in a girl with precocious puberty. *Journal of Neurosurgery*.

[18] O’Marcaigh A. S., Ledger G. A., Roche P. C., Parisi J. E., Zimmerman D. (1995). Aromatase expression in human germinomas with possible biological effects. *The Journal of Clinical Endocrinology & Metabolism*.

[19] Starzyk J., Starzyk B., Bartnik-Mikuta A., Urbanowicz W., Dziatkowiak H. (2001). Gonadotropin releasing hormone-independent precocious puberty in a 5 Year-old girl with suprasellar germ cell tumor secreting *β*-hCG and *α*-fetoprotein. *Journal of Pediatric Endocrinology and Metabolism*.

[20] De Sanctis V., Corrias A., Rizzo V. (2000). Etiology of central precocious puberty in males: the results of the Italian study group for physiopathology of puberty. *Journal of Pediatric Endocrinology and Metabolism*.

[21] Chemaitilly W., Merchant T. E., Li Z. (2016). Central precocious puberty following the diagnosis and treatment of paediatric cancer and central nervous system tumours: presentation and long-term outcomes. *Clinical Endocrinology*.

[22] Galifer R. B., Sultan C., Margueritte G., Barneo G. (1985). Testosterone-producing hepatoblastoma in a 3-year-old boy with precocious puberty. *Journal of Pediatric Surgery*.

[23] Anderson R. A., Mitchell R. T., Kelsey T. W., Spears N., Telfer E. E., Wallace W. H. B. (2015). Cancer treatment and gonadal function: experimental and established strategies for fertility preservation in children and young adults. *The Lancet Diabetes & Endocrinology*.

